# Hungarian, lazy, and biased: the role of analytic thinking and partisanship in fake news discernment on a Hungarian representative sample

**DOI:** 10.1038/s41598-022-26724-8

**Published:** 2023-01-05

**Authors:** Laura Faragó, Péter Krekó, Gábor Orosz

**Affiliations:** 1 Institute of Psychology, ELTE Eötvös Loránd University, Budapest, Hungary; 2 Political Capital Institute, Budapest, Hungary; 3 ULR 7369 -URePSSS - Unité de Recherche Pluridisciplinaire Sport Santé Société, Sherpas, Univ. Lille, Univ. Artois, Univ. Littoral Côte d’Opale, Liévin, France

**Keywords:** Human behaviour, Statistics

## Abstract

“Why do people believe blatantly inaccurate news headlines? Do we use our reasoning abilities to convince ourselves that statements that align with our ideology are true, or does reasoning allow us to effectively differentiate fake from real regardless of political ideology?” These were the questions of Pennycook and Rand (2019), and they are more than actual three years later in Eastern Europe (especially in Hungary) in the light of the rise of populism, and the ongoing war in Ukraine – with the flood of disinformation that follows. In this study, using a representative Hungarian sample (*N* = 991) we wanted to answer the same questions—moving one step forward and investigating alternative models. We aimed to extend the original research with the examination of digital literacy and source salience on media truth discernment. Most of the observations of Pennycook and Rand were confirmed: people with higher analytic thinking were better at discerning disinformation. However, the results are in line with the synergistic integrative model as partisanship interacted with cognitive reflection: anti-government voters used their analytic capacities to question both concordant and discordant fake news more than pro-government voters. Furthermore, digital literacy increased detection, but source salience did not matter when perceiving disinformation.

## Introduction

Most of the accumulated scientific knowledge on fake news derives from Western countries: the Anglo-Saxon World and Western Europe (for an overview, see e.g.,^1^). However, misinformation and disinformation present a major social, economic, and political problem in Eastern and Central Eastern Europe as well, with a more recently established and more fragile democratic institutional system, and the rise of illiberal populism. In Hungary—a country bordering Ukraine—60% of Hungarian respondents blamed Ukraine for the war initiated by Russia^2^, despite being invaded and occupied by Russia/the Soviet Union three times over the course of its history^3^. It seems like these strong anti-Ukrainian and pro-Russian views in Hungary are less related to historical experiences, and more to the fact that pro-government mainstream news outlets lead systematic disinformation campaigns and regularly broadcast pro-Kremlin propaganda^4–7^. The systematic conservative pro-government disinformation might be one of the tools that made Hungary a laboratory of illiberalism and post-truth indicating how European democratic values can decline, and attitudes can be shaped by a state-financed media conglomerate^6–8^. Hungary is becoming an “informational autocracy”, where the control of the social and political institutions is not exercised through oppression and violence, but the manipulation of information^6,9^ – which leads to a situation in which certain kinds of political fake news can have a stronger impact on pro-government voters. The goal of the present work is to re-examine the findings of Pennycook and Rand^10^ about the dominant role of “cognitive laziness” over biases (partisanship) on the ability to distinguish between real and fake news in a context of an informational autocracy. We were especially curious about the impact of cognitive reflection on the fake news discernment ability of pro-government voters in Hungary (in terms of distinguishing fake from real news).

Until most recently, two main models explained why people fall prey to fake news: the first is the “motivated system 2 reasoning” (MS2R^11^), and the second is the “classical reasoning theory”^10,12^. According to the first approach, the ability to think analytically (e.g., high scores on the cognitive reflection task, CRT^13^) increase partisan motivated reasoning. It means that people with a higher level of analytic thinking are more likely to believe in ideologically consistent misinformation and use their mental capacities to reinforce their already existing ideologies (see e.g.,^11,14,15^). Nevertheless, classical reasoning theory posits the complete opposite: not using mental capacities (e.g., being inattentive) is the main reason behind belief in fake news. According to this approach, analytic thinking decreases the perceived accuracy of fake news irrespective of its consistency with the previous ideologies^10,12,16–19^. Pennycook and Rand^10^ found no positive link between analytic thinking and belief in ideologically consistent fake news; therefore, in their interpretation, their results supported the classical reasoning theory over the motivated system 2 reasoning. A very recent study conducted in Ukraine provided further evidence for the classical reasoning theory even among Ukrainians who are strongly favoring Russia^17^.

Besides analytic thinking, the effect of specific partisanship (preference for a specific party) is not negligible. Pennycook and Rand^10^ found that conservative Trump voters are much more susceptible to misinformation than supporters of the democratic party. This result is in line with previous research about right-wing conservatives’ vulnerability to fake news^20–23^. However, the main statement of the classical reasoning theory appears to be true as both liberal and conservative analytic thinkers rated both politically concordant and discordant fake news headlines less accurate^10^.

Though there is empirical evidence supporting that the classical reasoning theory exceeds motivated system 2 reasoning, and cognitive reflection improves rather than decreases media truth discernment^10,12,16–19^, a new line of research aims to inherently integrate the relevance of partisanship into the mechanisms of analytic thinking. According to this integrative model, partisanship, identity-protective thinking, and analytic thinking are all important predictors of belief in misinformation^24–26^. For instance, Rozenbeek et al.^24^ found in a US sample that compared to analytic thinking, political liberalism was more strongly related to identifying fake news. These results are not against the classical reasoning theory, but they put the relative importance of analytic thinking into a new perspective. In the present study, our goal is to replicate the results of Pennycook and Rand^10^, and also to go beyond the comparison of the MSR2 account and the classical reasoning theory by testing predictions deriving from the integrative model in the Hungarian context, where disinformation is mainstream in the public media.

Above the replication of Study 1 of Pennycook and Rand^10^, we also aim to investigate the effect of digital literacy and the salience of the source on the perceived accuracy of real and fake news. Previous research found contradictory results regarding digital literacy: some authors suggested that those who competently use social media and the Internet are less likely to believe in fake news (e.g., ^27,28^), but other scholars argue that only information literacy (the ability to search for reliable information) predicts accurate beliefs, while other types of media literacy do not^29^. The contradiction is even greater regarding the salience of the source: some studies found that the credibility of the news source increases the recognition of fake news (e.g.,^18^), but other scholars claim that the source of news is not an important predictor of fake news acceptance^12,30,31^, even though users rate mainstream news sources more trustworthy than partisan or fake sources^32^. Consequently, we aim to investigate whether digital literacy and the salience of the source increase media truth discernment in this cultural context.

### The current study and hypotheses

In this replication, based on the results of Pennycook and Rand^10^, we presumed that analytic thinking would reduce fake news accuracy ratings (trueness of fake news) and increase media truth discernment (distinguishing fake from real news; see also^12,16–19^) (H1). We also expected that analytic thinking would not increase partisan motivated reasoning of politically consistent fake news items. In other words, we presumed the lack of positive correlations between analytic thinking and accuracy ratings of politically concordant fake news (H2). If deliberative thinking would increase motivated reasoning as it is hypothesized by the motivated system 2 reasoning account^11^, then correlations between the perceived accuracy of politically consistent fake news items and analytic thinking should be positive.

We also hypothesized based on Pennycook and Rand^10^ that participants will be better able to distinguish real from fake news if the news content is politically consistent with their pre-existing political preferences (vs. inconsistent), so the difference in the perceived accuracy of fake and real news will be significantly larger for politically concordant headlines than for politically discordant headlines (H3).

Besides the above-mentioned main goals, we also expected that voters of the conservative Hungarian government party would be worse at distinguishing fake from real news than supporters of opposition parties, based on the previous research^6,10,20–23^ (H4). To test the integrative approach^24–26^, we presumed that opposition voters would benefit more from analytic thinking in terms of distinguishing fake from real news compared to pro-government voters (H5). We also hypothesized based on the prediction of van der Linden^26^ that political fake news will be better explained by partisanship than by analytic thinking, while non-political misinformation is predicted better by analytic thinking, in line with the integrative approach (H6).

As an extension of the original study, fake and real news headlines were given to half of the participants either with the source presented on the headline or with the source completely omitted. We hypothesize that those who received the headlines with the source indicated will be more likely to discern real from fake news than those who did not see the source (in line with^18^) (H7). In addition to the impact of the salience of the source, we presume that those who have higher digital literacy skills will be far better at discerning real news from disinformation than people low on digital competencies^27,28^ (H8).

## Methods

### Participants

We collected data via an online survey (in Qualtrics). The collection of the sample was administered by a Hungarian polling company (Medián). Our probability sample was representative in terms of age, gender, and region of those Hungarian adults who use the internet at least once a week. Respondents were randomly selected from two internet-enabled panels with 360,000 and 160,000 members respectively, and the sample was created using a multiple-step, proportionally stratified, probabilistic sampling method. Members are automatically excluded from the panels if they have an inactive or incorrect email address.

We did not conduct an a-priori power analysis to determine the sample size but aimed at *N* = 1,000 which is generally used in pollster surveys relying on representative samples of Hungarian society. Our final sample consisted of 991 respondents with a mean age of 50.23 (*SD*_*age*_ = 16.07, aged between 18 and 91 years) and 52.1% of the respondents were female. Regarding the highest level of education, 46.8% had a high-school degree, and 32.5% received a post-secondary degree. The proportion of pro-government voters was 38.6%. The date of data collection was April 2021. The research was conducted with the IRB approval of Eötvös Loránd University in accordance with the Declaration of Helsinki, and with the informed consent of the participants.

### Materials and procedure

Similarly to the protocol of Pennycook & Rand^10^, participants were presented with 15 factually fake and 15 real news headlines, all of which were published in Hungarian fact-checking sites or mainstream news sources. From the 15 fake and 15 real headlines, five real and five fake were pro-Orbán (ideologically consistent for conservative pro-government voters [in parallel with Republican-consistent news], e.g., *“We [Hungary] will be among the top 5 countries after the migration crisis has subsided”*), five real and five fake were anti-Orbán (worldview-consistent for supporters of the opposition [in parallel with Democrat-consistent news], e.g., *"We got twenty thousand forints [50USD] per person to get on the bus [from abroad] and come to Hungary to listen to Orbán's speech [in the capital]*), and five real and five fake were politically neutral (e.g., *“Telepathy exists and anyone can develop this ability”*). The news resembled a Facebook post including a picture, a headline, and a byline, and was randomly presented to participants. All headlines were pre-tested before the data collection.

We asked participants regarding each headline whether they have seen or heard about the story before (no/unsure/yes); to their best knowledge, how accurate is the claim in the headline (not at all accurate/not very accurate/somewhat accurate/very accurate); and whether they would consider sharing the story online (I would never share something political online/no/maybe/yes)^10^.

After evaluating the 30 headlines, respondents were given the 7-item cognitive reflection test used in the original study of Pennycook & Rand^10^ (e.g., *A farmer had 15 sheep and all but 8 died. How many are left? (intuitive answer: 7; correct answer: 8)*^13,33,34^. Responses were coded as right (1) or wrong (0), and we created the mean from the items (the higher the mean, the more analytically a person thinks). The scale has similarly good internal consistency as in the original study (Cronbach’s α = 0.75).

After the cognitive reflection tasks, respondents completed a digital media literacy test (e.g., *“I rely on family members to introduce me to new technology”, response options: very often/somewhat often/occasionally/almost never/never*)^35^, which also had good internal consistency (Cronbach’s α = 0.73).

Demographic variables (age, gender, type of settlement, highest level of education, party preference) were administered at the end of the survey.

### Analytic strategy

We used and analyzed only the perceived accuracy of the headline in further analyses and calculated the means separately for the perceived accuracy of fake and real news items (the higher the mean, the higher the perceived accuracy). To calculate media truth discernment scores, we created z-scores from the perceived accuracy of real and fake news and subtracted the z-score of fake news from the z-score of real news (see ^10,19^). We aimed to strictly follow the analytic strategy of Pennycook and Rand^10^ by using Pearson correlations and ANOVAs. Finally, to test the integrative model, we added supplemental analyses to examine the moderating effect of partisanship on the link between analytic thinking and media truth discernment, and we implemented an OLS regression. We conducted Pearson correlations and z-tests to investigate predictors of political and non-political disinformation. IBM SPSS software version 28.0 and R 4.0.3^36^ were used for statistical analyses and data visualization.

## Results

First, by using the OLS regression, we tested the relationship between analytic thinking, partisanship, digital literacy, source salience, and the distinction between real and fake news while controlling for demographic variables (the residuals were not correlated (Durbin-Watson = 1.667), and no VIF value exceeded 1.2). Beyond demographic variables (gender [*ß* = -0.06, *p* = 0.072], level of education [*ß* = 0.18, *p* = 0.001], and age [*ß* = -0.06, *p* = 0.052]), media truth discernment (real news’ mean accuracy rating minus fake news’ mean accuracy rating) was predicted significantly by partisanship (anti-government higher, *ß* = 0.23, *p* = 0.001), by analytic thinking (higher, *ß* = 0.15, *p* = 0.001), and by digital media literacy (higher, *ß* = 0.12, *p* < 0.001), but was not predicted significantly by the salience of the source (*ß* = 0.04, *p* < 0.138). Therefore, partisanship, analytic thinking, and digital media literacy were related to distinguishing real from fake news (even if we consider relevant sociodemographic variables).

The correlation between media truth discernment and analytic thinking (*r* = 0.27, *p* = 0.001) did not differ significantly from the correlation between media truth discernment and partisanship (*r* = 0.26, *p* = 0.001) (*z* = 0.25, *p* = 0.402), from the correlation between education level and media truth discernment (*r* = 0.24, *p* = 0.001) (*z* = 0.83, *p* = 0.204), and from the correlation between digital literacy and media truth discernment (*r* = 0.21, *p* = 0.001) (*z* = 1.56, *p* = 0.06), so these factors predicted media truth discernment with similar strength.

Using Pearson correlations, we found that analytic thinking (in terms of higher cognitive reflection scores) was negatively related to rating fake news correctly (*r* = -0.18, *p* = 0.001) and was positively related to the accuracy rating of real news (*r* = 0.08, *p* = 0.012), even if the correlations were small. Therefore, our results suggest that stronger analytic thinking is associated with more precise and accurate judgments about real and fake news. Furthermore, digital media literacy can help people to accurately recognize fake news (*r* = -0.17, *p* < 0.001), but it does not necessarily promote the recognition of real news (*r* = 0.02, *p* = 0.44). Nevertheless, the salience of the source of the news (source indicated vs. not indicated) did not have any impact on media truth discernment. It appears that indicating the news sources will not help the recognition of the fake news.

Descriptive statistics of the main variables can be found online in the Supplementary Material (Table S1).

### Analytic thinking, partisanship, and politically concordant vs. discordant news content

Similar to Pennycook and Rand^10^, no positive correlations were found between analytic thinking and accuracy ratings of politically consistent fake news. This means that analytic thinking was not related to the accuracy ratings of politically congruent fake news (see Table 1). Instead, analytic thinkers (people scoring high on the cognitive reflection task) rated both politically consistent and inconsistent fake news headlines as less accurate and it was also true for both pro- and anti-government voters. It means that analytic thinking can be protective from disinformation irrespectively from the nature of news content (concordant vs. discordant) and irrespectively from partisanship (voting for the government vs. the opposition). The association of CRT and partisanship on the accurate evaluation of political fake and real news headlines is presented online in the Supplementary Material (Figure S1a and Figure S1b).

**Table 1:** Correlation between CRT and the accuracy of judgments.

	Pro-Orbán news (~ Republican-consistent news in the original work)			Anti-Orbán news (~ Democrat-consistent news in the original work)			Non-political news		
	Fake news	Real news	Real-fake news	Fake news	Real news	Real-fake news	Fake news	Real news	Real-fake news
Pro-government voters	− 0.02	0.07	0.10*	− 0.01	0.07	0.08	− 0.18**	0.05	0.22**
Anti-government voters	− 0.27**	− 0.04	0.23**	− 0.09*	0.06	0.14**	− 0.23**	0.09*	0.31**

Real-Fake News = Media truth discernment. Pro-Orbán news = Pro-government news, Anti-Orbán news = Anti-government news. We did not use both the expressions of pro-government / anti-government news and pro-government / anti-government voters at the same time for the sake of clarity and easy understanding. Negative correlations in the fake and real columns mean that the more analytic people are, the less accurate they find that specific type of news. Positive correlations in the media truth discernment column indicate that the more analytic individuals are, the better they can distinguish fake news from real ones. Statistical significance is indicated at the following levels: ***p < 0.001; **p < 0.01; *p < 0.05.

The salience of the source of the news (source indicated vs. not indicated) did not have any impact on accuracy ratings and the CRT-accuracy correlations, neither in terms of main effects nor interactions in any of the analyses, therefore, we decided to merge these two conditions in further analyses.

### Political concordance of news content and fake vs. real news accuracy ratings without analytic thinking

**Figure 1:**
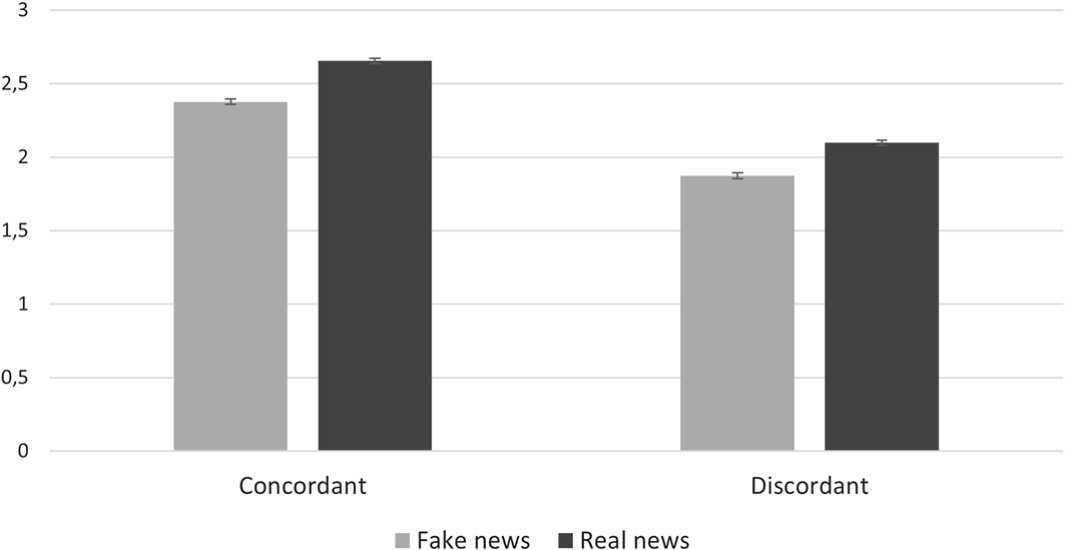
Mean perceived accuracy of fake and real news headlines as a function of political concordance. This result suggests that participants were better able to distinguish real from fake news if the news content was politically consistent with their pre-existing political preferences (vs. inconsistent). Error bars represent 95% CIs. *Note.* Concordant = pro-Orbán (~ Republican-consistent news in the original work) headlines for pro-government voters and anti-Orbán headlines for voters of the opposition; Discordant = anti-Orbán (~ Democrat-consistent news in the original work) headlines for pro-government voters and pro-Orbán headlines for voters of the opposition).

A post hoc analysis revealed a significant interaction between political concordance and fake and real new accuracy ratings, *F*(1,990) = 5.158, *p* = 0.023, *ƞ*^*2*^ = 0.005 (see Fig. 1). It means that, as in the original paper, the difference in the perceived accuracy of fake and real news was significantly larger for politically concordant headlines than for politically discordant headlines. This result (in line with the findings of Pennycook and Rand^10^) suggests that participants were better able to distinguish real from fake news if the news content was politically consistent with their pre-existing political preferences (vs. inconsistent), *t*(990) = 2.27, *p* = 0.023, *d* = 0.35.

**Figure 2:**
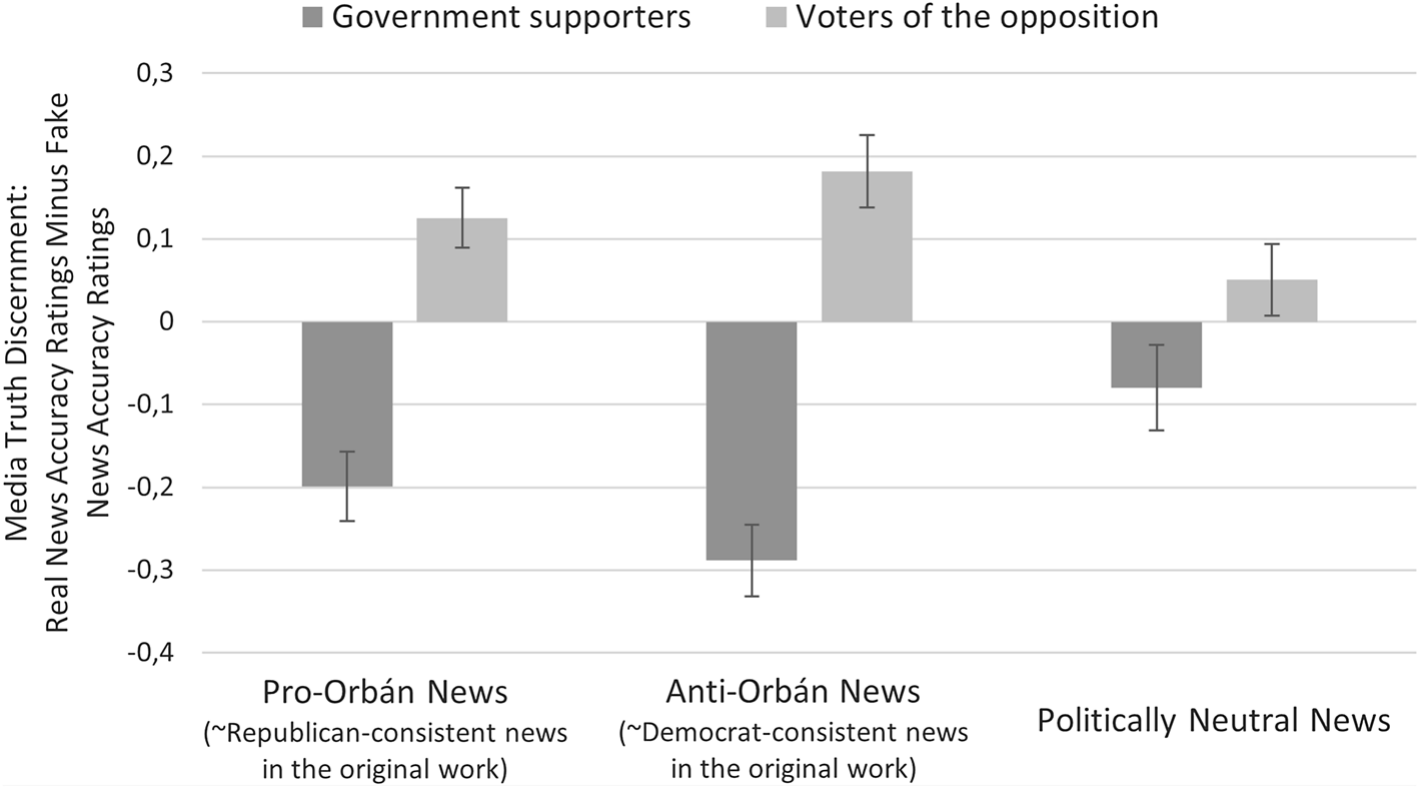
Media truth discernment as a function of political ideology and political leaning of the headline. Supporters of the opposition were considerably better at discerning fake from real news (irrespective of the type of news) than supporters of the government. Error bars represent 95% CIs. *Note.* High media truth discernment score indicates an increased capacity to distinguish real from fake news. “Government supporters” is rather similar to “Republican voters” in the context of the original work (and the US context) while “Voters of the opposition” is an analogy with “Democrat voters” in the Trump era.

Besides separate real and fake news accuracy ratings, following the original work of Pennycook and Rand^10^, we were also interested in how media truth discernment is shaped by partisanship and the political leaning of the headline. We conducted the same mixed-design ANOVA with the political leaning of the headline (pro-Orbán, anti-Orbán, politically neutral) as a within-subject factor and partisanship (pro-government vs. opposition voters) as a between-subject factor. The results are seen in Fig. 2.

Specific partisanship had a significant main effect on media truth discernment scores *F*(1,989) = 48.69, *p* = 0.001, *ƞ*^*2*^ = 0.05, similar to the results of Pennycook and Rand (*ƞ*^*2*^ = 0.04). It means that supporters of the opposition were significantly better at discerning fake from real news (irrespective of the type of news) than pro-government supporters. There was no main effect of type of news, *F*(2,988) = 0.43, *p* = 0.649, *ƞ*^*2*^ = 0.001, nevertheless, we revealed a significant interaction effect between partisanship and type of news *F*(2,988) = 8.4, *p* = 0.001, *ƞ*^*2*^ = 0.02, consistent with the result of Pennycook and Rand. Supporters of the opposition were far better at discerning anti-Orbán real news from disinformation than voters of the governing party *t*(942.2) = − 7.66, *p* = 0.001, *d* = − 0.47.

Nevertheless, in contrast to the original results, there was a significant difference in the discernment of pro-Orbán fake and real news based on partisanship *t*(989) = − 5.77, *p* = 0.001, *d* = 0.38: supporters of the opposition were significantly better at recognizing their discordant, pro-Orbán fake news than voters of the governing party. Pennycook and Rand^10^ also found this difference between Clinton- and Trump supporters regarding pro-Trump news, but that difference was insignificant. Nonetheless, anti-government voters were not significantly better at discerning politically neutral real news from misinformation than supporters of the government *t*(989) = − 1.91, *p* = 0.056, *d* = 0.13.

Despite the cultural differences, all in all, the bulk of our results confirms the findings of Pennycook and Rand^10^ and supports the classical reasoning theory rather than the motivated system 2 reasoning account. In the following, we would like to demonstrate some additional analyses which support the integrative model and highlight some relevant cultural differences that might be related to the effect of a decade-long projection of disinformation in the mainstream public media which is absent in the US.

### Disparity in analytic thinking between pro- and anti-government voters and its relationship with media truth discernment

**Figure 3:**
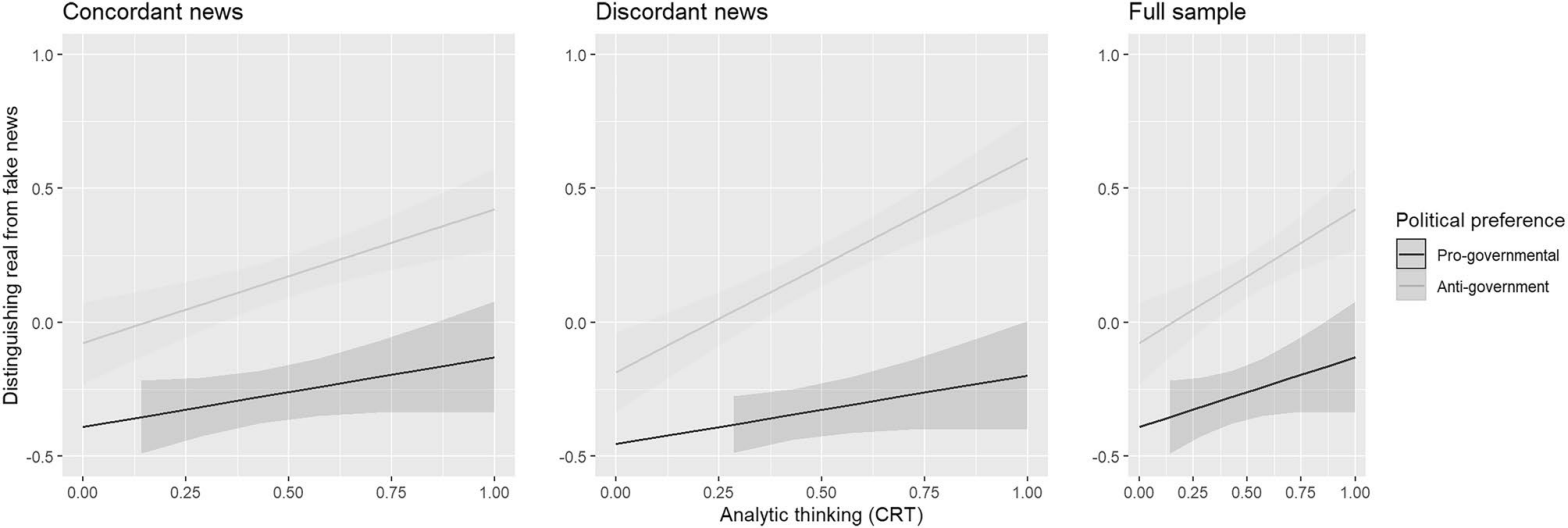
The interaction between analytic thinking and partisanship on media truth discernment for concordant news, discordant news, and for the full sample (including all types of news). The result suggests that anti-government voters can benefit more from analytic thinking in terms of distinguishing fake from real news compared to pro-government voters. *Note.* Analytic thinking (x-axis): 0 = minimal score of analytic thinking; 1 = maximal score of analytic thinking. Distinguishing real from fake news = Media truth discernment. On the y-axis, low values indicate the correctness of fake news with incorrect ratings of real news, while high values indicate the perceived inaccuracy of fake news with correct ratings of real news. Negative values mean that people found fake news more accurate than real news. Positive values indicate that real news correctness ratings are higher than fake news correctness ratings.

We ran an additional analysis in which we were interested in whether pro- vs. anti-government voters benefit more from analytic thinking in the accuracy ratings of fake news (including all sorts of news). All in all, the results suggest that besides the main effects of analytic thinking, *t*(987) = 3.14, *p* = 0.002, *d* = 0.15, and partisanship, *t*(987) = 8.03, *p* = 0.001, *d* = 0.49, anti-government voters can benefit more from analytic thinking in terms of distinguishing fake from real news compared to pro-government voters, *t*(987) = 2.53, *p* = 0.012, *d* = 0.16, see Fig. 3.

### Predictors of political and non-political disinformation

To further test the integrative model, we investigated the prediction of van der Linden^26^ that political fake news will be better explained by partisanship than by analytic thinking, while non-political misinformation is predicted better by analytic thinking. Indeed, regarding political fake news, the correlation between media truth discernment and partisanship (*r* = 0.30, *p* = 0.001) differed significantly from the correlation between media truth discernment and analytic thinking (*r* = 0.21, *p* = 0.001) (*z* = 2.23, *p* = 0.013), indicating that partisanship is a stronger correlate of political disinformation than analytic thinking. However, regarding non-political fake news, the correlation between media truth discernment and analytic thinking (*r* = 0.28, *p* = 0.001) proved to be stronger than the correlation between media truth discernment and partisanship (*r* = 0.06, *p* = 0.056) (*z* = 5.32, *p* = 0.000), showing that analytic thinking is associated with belief in non-political misinformation, while partisanship is not.

## Discussion

### Theoretical implications

The present study aimed to replicate Study 1 of Pennycook & Rand^10^ on a representative Hungarian sample, and our results suggest that the classical reasoning theory indeed better explains the (lack of) ability to discern real from fake news than the motivated system 2 reasoning account. Analytic thinking was related positively to media truth discernment in line with the previous research^10,12,16–19^, meaning that those Hungarians who think deliberately are more likely to recognize fake information. Furthermore, similarly to Pennycook and Rand^10^, we did not find a positive relationship between analytic thinking and ideologically concordant fake news headlines. Thus, deliberate thinking did not increase partisan motivated reasoning contrary to previous research supporting the motivated system 2 reasoning account^11,14,15^. This was true regardless of respondents’ partisanship and the ideological concordance of the news content: analytic supporters of both the government and the opposition were more likely to identify fake information as inaccurate and it was true for ideologically matching and mismatching news headlines. We also found a significant interaction between the political concordance of the headline and the type of news (whether it is fake or real), but the effect was visibly weaker than in the original study. This result suggests that respondents are better at discerning real from fake news which is worldview-consistent rather than inconsistent.

Nevertheless, in Hungary, asymmetric partisanship and partisan motivated reasoning are equally important, even more important than the liberal-conservative, or the leftist-rightist distinction (see e.g.,^37^). In this cultural context, though analytic thinking increases media truth discernment both for supporters of the government and the opposition, partisanship influences the level of analytic thinking (with higher analytic thinking on the opposition side), and the two affect the recognition of disinformation in interaction. Specifically, analytic thinking increased fake news discernment more on the opposition, than on the governmental side. Therefore, our results support rather the integrative model^24–26^ than the classical reasoning account, and we can posit that both specific partisanship and analytic thinking are important predictors of media truth discernment. Our results are in line with the study of Borukhson et al.^38^, who found using computational modeling that the integrative model outperformed both the MS2R account and the classical reasoning theory in explaining susceptibility to disinformation. Furthermore, political fake news was better predicted by partisanship than by analytic thinking in our study, which further supports the integrative approach^24–26^. To understand the interaction between partisanship and analytic thinking—that is compatible with the integrative model—we can turn to the applied implications.

### Applied implications

Besides the replication analyses, we also investigated the interaction between analytic thinking and partisanship (supporting the governing party vs. the opposition). According to these results, anti-government voters could benefit more from analytic thinking in terms of distinguishing fake from real news compared to pro-government voters and it is especially true for ideologically discordant news content. In contrast to the supporters of the conservative government, Hungarian opposition voters appear to be less vulnerable to disinformation and they could distinguish fake from real news independently from the news content’ ideologically matching or mismatching nature.

These results can be explained by the specific Hungarian political and societal context. The government gradually obtained most of the offline and online media outlets over the past 12 years. These pro-government news outlets became mainstream and have been disseminating pro-Kremlin and anti-Western disinformation^4–7^. These long years of disinformation exposure could have had an in-depth effect on pro-government voters’ ability to recognize disinformation effectively compared to voters of the opposition who tried to follow the dynamically narrowing set of anti-government and liberal news sources.

Apart from the government-induced systematic and broad-scale disinformation campaign of the governmental news outlets, the socially conservative attitudes of the pro-government voters could also play a role. Voters have heightened susceptibility to threats and negative information^21,23^, which might explain one of the reasons why they find disinformation with threatening content more credible. There is also ample evidence suggesting that conservative voters have a lower need for cognition and a lack of motivation to think critically^22^. For instance, social conservatives were more likely to accept sentences that look sublime but are meaningless, than liberals^39–42^ (for an exception, see^43^). Their receptivity to "bullshit" sentences is positively correlated with intuitive thinking and fake news accuracy^12^. According to Jost^22^, using the same cognitive reflection test we used in the present study to assess analytic thinking, prior studies consecutively showed a marked difference between conservatives and liberals: 11 studies showed liberals perform better on the test, and only one study showed no difference along partisanship. In our study, pro-government voters had significantly lower analytic thinking (*M* = 0.44, *SD* = 0.30) than anti-government voters (*M* = 0.50, *SD* = 0.30, *t*(989) = − 3.47, *p* = 0.001, *d* = − 0.23), so it appears that pro-government voters relied much less on reflective thinking than those who would vote for any of the opposition parties^22,44,45^. However, it is not related to individual differences in intelligence, but rather the lack of motivation to use intellectual capacities on the conservative side of the political spectrum. Thus, in the Hungarian context, the striking difference between pro-government vs. anti-government voters might be related to the decade-long propaganda that made pro-government voters less motivated to use their cognitive capacities to distinguish fake from real news.

Regarding the additional analyzes, our results support that digital literacy skills indeed positively predict media truth discernment in line with^27,28^, contrary to^29^. Thus, the more a person is knowledgeable about basic terms of the internet and social media, the more they can identify fake news. Nevertheless, the salience of the source of news did not help individuals to recognize disinformation: whether the source was included or omitted from the headline neither affected the regressions nor interacted with other variables in the analyses. This finding is in line with^12,30,31^ but contradicts the results of^18^. This suggests that respondents were more likely to focus on the picture, the headline, and the byline of the news, so on the content, and the heuristic of the reliability of the source of news did not play a role in their decision when they evaluated the perceived accuracy of fake and real news.

### Limitations and future directions

This study is not without limitations. First, our data collection started amid the COVID-19 waves, and despite preparing a preregistration document for our study, we did not upload it due to an administrative error before data collection. Second, though we followed the methodological guidelines of Pennycook and Rand^10^, the stimulus set was not as completely realistic as reading news in a real-life context, e.g., when one is browsing their social media timeline. Though the headlines were similar to those that are found on social media, they were not embedded in their original online environment, which might have affected the ecological validity of our study. Third, we examined the same general psychological mechanisms as Pennycook & Rand^10^ in this specific Hungarian information environment and found very similar results. However, we do not know whether these results can be extrapolated to other Eastern European countries.

In future studies, one might investigate the contextual moderators that can strengthen or weaken the general effect of analytic thinking on perceived news accuracy. Among these contextual elements, further investigation to separate the impact of partisanship and media consumption is required. Partisanship should be examined both from the perspective of voting intentions (government vs. opposition support) and political identity (political conservatism vs. liberalism). Online and offline media consumption habits also appear to matter and should be examined in the context of the systematic disinformation campaigns of governmental news outlets. Finally, future intervention studies might examine how to make conservative pro-government supporters more motivated to use their cognitive capacities to recognize disinformation.

## Conclusion

We managed to replicate most results of Pennycook and Rand^10^, namely that people with a higher level of analytic thinking were better at discerning real from fake news, and deliberative thinking did not increase partisan motivated reasoning, in line with the classical reasoning theory. Nonetheless, we claim that the integrative model explains better the Hungarian results than purely classical reasoning as partisanship interacted with analytic thinking. It means that anti-government voters used their analytic capacities more to question fake news than supporters of the conservative government. We can conclude that Hungarian pro-government voters have a hard time differentiating between real and fake news. One possible solution might be utilizing digital media literacy competency development programs that can promote beneficial strategies for using cognitive capacities to identify fake news. Unfortunately, Hungarian students scored significantly lower than the OECD average in each category on the PISA tests with a steep decline from 2009 (see^46^) and Hungarian teachers’ salaries are among the lowest in the OECD^47^. These numbers might suggest that the Hungarian government does not seem to invest in the improvement of general and digital cognitive capacities that could help Hungarian citizens to distinguish fake from real news.

## Supplementary Information

Supplementary Information.

## Data Availability

The dataset that supports the findings (The Role of Analytic Thinking, Partisanship, Digital Literacy, and Source Salience in Fake News Discernment on a Hungarian Sample.sav) is openly available on Open Science Framework (https://doi.org/10.17605/OSF.IO/VCF36) and can be found here: https://osf.io/4r29u.
