# Supplementary material for: Hungarian, lazy, and biased: the role of analytic thinking and partisanship in fake news discernment on a Hungarian representative sample
Source: Sci Rep. 2023 Jan 5;13:178. doi: 10.1038/s41598-022-26724-8 (PMC9813452; doi:10.1038/s41598-022-26724-8)
Supplement: Supplementary file 1 — Supplementary Information. [file 41598_2022_26724_MOESM1_ESM.docx]

**Supplementary Materials**

**for**

**Hungarian, Lazy, and Biased: The Role of Analytic Thinking and Partisanship in Fake News Discernment on a Hungarian Representative Sample**

Content

**Data sharing policy2**

**Table S1: Descriptive statistics of main variables3**

**Figure S1a: Mean accuracy ratings as a function of partisanship and CRT for fake news4**

**Figure S1b: Mean accuracy ratings as a function of partisanship and CRT for real news5**

**Data sharing policy**

The dataset that supports the findings (*The Role of Analytic Thinking, Partisanship, Digital Literacy, and Source Salience in Fake News Discernment on a Hungarian Sample.sav*) is openly available on Open Science Framework (DOI 10.17605/OSF.IO/VCF36) and can be found here: https://osf.io/4r29u.

**Table S1: Descriptive statistics of main variables**

|  | *N = 991* | |  |
| --- | --- | --- | --- |
|  | *M* | *SD* |  |
| Accuracy of pro-Orbán fake news | 1.97 | 0.66 |  |
| Accuracy of pro-Orbán real news | 2.22 | 0.59 |  |
| Accuracy of anti-Orbán fake news | 2.28 | 0.63 |  |
| Accuracy of anti-Orbán real news | | 2.54 | 0.70 |
| Accuracy of politically neutral fake news | 1.90 | 0.53 |  |
| Accuracy of politically neutral real news | 2.37 | 0.56 |  |
| Cognitive reflection test | 0.48 | 0.30 |  |
| Digital media literacy | 3.47 | 0.71 |  |

*Note:* The acceptance of fake and real news was measured with a scale from 1 (not at all accurate)
to 4 (very accurate), higher means indicate higher perceived accuracy. Cognitive reflection items were coded as correct (1) and wrong (0), a higher mean represents higher analytical thinking. Digital media literacy was measured with a scale ranging from 1 (very often) to 5 (never), a higher mean indicates higher digital media literacy.

The salience of the source of the news (source indicated vs. not indicated) did not have any impact on accuracy ratings (similar to Pennycook & Rand, 2020), therefore, we decided to merge the two conditions in descriptive statistics and further analyses.

**Figure S1a. Mean accuracy ratings as a function of partisanship and CRT for fake news.** These results suggest that the more analytical individuals are, the better they recognize fake news, regardless of respondents’ partisanship and the type of news.

*Note.* Error bars represent 95% CIs. The figure strictly follows Pennycook and Rand’s (2019) protocol of demonstration of these results. Intuitive = lowest 25%, highest 25% of cognitive reflection task scores (analytic thinking)

**Figure S1b. Mean accuracy ratings as a function of partisanship and CRT for real news.** These results suggest that the more analytical individuals are, the better they recognize real news, regardless of respondents’ partisanship and the type of news. The only exception is the pro-Orbán real news for anti-government voters.

*Note.* Error bars represent 95% CIs. The figure strictly follows Pennycook and Rand’s (2019) protocol of demonstration of these results. Intuitive = lowest 25%, highest 25% of cognitive reflection task scores (analytic thinking)
